# Whole Genome SNP Genotyping and Exome Sequencing Reveal Novel Genetic Variants and Putative Causative Genes in Congenital Hyperinsulinism

**DOI:** 10.1371/journal.pone.0068740

**Published:** 2013-07-15

**Authors:** Maria Carla Proverbio, Eleonora Mangano, Alessandra Gessi, Roberta Bordoni, Roberta Spinelli, Rosanna Asselta, Paola Sogno Valin, Stefania Di Candia, Ilaria Zamproni, Cecilia Diceglie, Stefano Mora, Manuela Caruso-Nicoletti, Alessandro Salvatoni, Gianluca De Bellis, Cristina Battaglia

**Affiliations:** 1 Dipartimento di Fisiopatologia e dei Trapianti (DePT), Università degli Studi di Milano, Milan, Italy; 2 Institute of Biomedical Technologies (ITB), CNR, Segrate, Milan, Italy; 3 Scuola di Dottorato di Medicina Molecolare, Università degli Studi di Milano, Milan, Italy; 4 Dipartimento di Biotecnologie Mediche e Medicina Traslazionale (BIOMETRA), Università degli Studi di Milano, Milan, Italy; 5 Department of Pediatrics, San Raffaele Scientific Institute, Milan, Italy; 6 Laboratory of Pediatric Endocrinology, Division of Metabolic and Cardiovascular Sciences, San Raffaele Scientific Institute, Milan, Italy; 7 Dipartimento di Scienze Mediche e Pediatriche, Università di Catania, Catania, Italy; 8 Department of Clinical and Experimental Medicine, Pediatric Unit, Insubria University, Varese, Italy; University of Alabama at Birmingham, United States of America

## Abstract

Congenital hyperinsulinism of infancy (CHI) is a rare disorder characterized by severe hypoglycemia due to inappropriate insulin secretion. The genetic causes of CHI have been found in genes regulating insulin secretion from pancreatic β-cells; recessive inactivating mutations in the *ABCC8* and *KCNJ11* genes represent the most common events. Despite the advances in understanding the molecular pathogenesis of CHI, specific genetic determinants in about 50 % of the CHI patients remain unknown, suggesting additional locus heterogeneity. In order to search for novel loci contributing to the pathogenesis of CHI, we combined a family-based association study, using the transmission disequilibrium test on 17 CHI patients lacking mutations in *ABCC8*/*KCNJ11,* with a whole-exome sequencing analysis performed on 10 probands. This strategy allowed the identification of the potential causative mutations in genes implicated in the regulation of insulin secretion such as transmembrane proteins (*CACNA1A, KCNH6, KCNJ10, NOTCH2, RYR3, SCN8A, TRPV3, TRPC5*), cytosolic (*ACACB, CAMK2D, CDKAL1, GNAS, NOS2, PDE4C, PIK3R3*) and mitochondrial enzymes (*PC, SLC24A6*), and in four genes (*CSMD1*, *SLC37A3*, *SULF1*, *TLL1*) suggested by TDT family-based association study. Moreover, the exome-sequencing approach resulted to be an efficient diagnostic tool for CHI, allowing the identification of mutations in three causative CHI genes (*ABCC8*, *GLUD1, and HNF1A)* in four out of 10 patients. Overall, the present study should be considered as a starting point to design further investigations: our results might indeed contribute to meta-analysis studies, aimed at the identification/confirmation of novel causative or modifier genes.

## Introduction

Congenital hyperinsulinism (CHI), previously known as persistent hyperinsulinemic hypoglycemia of infancy (PHHI, MIM256450), is characterized by severe hypoglycemia due to inappropriate insulin secretion from pancreatic β-cells. If improperly managed, hypoglycemia can cause brain damage, learning disability, and even death [1]. This condition affects at least 1/50,000 children of European descent, and it has been reported in nearly all major ethnic groups [2].

Histologically, CHI can be associated either with diffuse insulin secretion or with focal adenomatous hyperplasia. These two forms share a similar clinical presentation, but result from different molecular mechanisms. Recently, a positron emission tomography scan using Fluorine-18 L-3,4-dihydroxyphenylalanine (18-fluoro DOPA-TC-PET-scan) has been used to distinguish focal from diffuse forms. Diffuse CHI (Di-CHI) is characterized by autosomal recessive or (less frequently) dominant inheritance, whereas focal CHI (Fo-CHI) is due to a germline paternal mutation (in the *ABCC8 or KCNJ11* gene) in addition to a somatic loss of the maternally-derived chromosome 11p15.1 region in pancreatic β-cells [2]. According to the molecular defects, CHI has been classified as a channelopathy (K_ATP_ channel), as a metabolic enzyme/transporter anomaly, or as a transcription factor defect [1]. Medical treatments of CHI include different drugs such as oral diazoxide (channel activator), somatostatin analogue (octreotide) injections, and appropriate diet. The surgical treatment with subtotal pancreatectomy is required in Di-CHI when medical treatment and dietary therapies are ineffective, whereas Fo-CHI can be cured with resection of the focal area of adenomatous hyperplasia. In the first case, the long-term outcome is characterized by the risk of developing diabetes.

Overall, CHI causative mutations have been documented in 10 genes, allowing a molecular diagnosis for nearly 50% of CHI patients [3], [4]. The most common molecular cause of CHI is the dysfunction of the pancreatic K^+^_ATP_ channel encoded by the sulfonylurea receptor gene (*SUR1*, alias *ABCC8*) and the inward-rectifying potassium channel gene (*KIR6.2*, alias *KCNJ11*). Less frequently, recessive mutations in the *HADH* gene (3-hydroxyacyl-Coenzyme A dehydrogenase) have been reported. Dominant forms of CHI are due to either activating mutations in mitochondrial matrix enzymes, *GLUD1* (glutamate dehydrogenase 1) and *UCP2* (mitochondrial uncoupling protein 2), or to mutations in *HNF4A* (hepatocyte nuclear factor 4 alpha) and *SLC16A1* (monocarboxylate transporter 1). Activating mutations in the *GCK* (glucokinase) may also lead to CHI, and an autosomal dominant mutation in *INSR* (insulin receptor gene) has been described in a large Danish pedigree [5]. Very recently, mutations in the *HNF1A* (hepatocyte nuclear factor 1 alpha) gene have been reported in CHI patients [6], [7].

Insulin secretion is regulated by a complex network of proteins [8] acting at the cellular membrane level (i.e. ATP sensitive K^+^ channels, Ca^2+^, Na^+^ voltage-gated channels, glucose transporters, and transient receptors) [9], at the intracellular level (i.e. endoplasmic reticulum ion channels), and at the mitochondrial level (i.e. enzymes) [10]. Since CHI is characterized by severe hypoglycemia due to inappropriate insulin secretion from pancreatic β-cells, we hypothesized that mutations in genes involved in this process might be responsible for the disease. Moreover, considering the phenotypic variability observed within CHI families, “phenotypic modifier” genes might contribute to the global genetic picture of this disorder as described for other Mendelian disorders [11].

The mutational screening of CHI causative genes is usually carried out by PCR amplifications and direct Sanger sequencing, using an iterative approach based on clinical features and/or on medical treatment response [2].

High-density single nucleotide polymorphism (SNP)-array technology has been widely used to perform linkage analysis, including the transmission-disequilibrium test (TDT). Moreover, the combination of linkage analysis with haplotype information (applied to trios) has been successful applied to map Mendelian traits [12] and has led to the identification of gene variants causing diseases and birth defects [13]. Recent developments in high-throughput sequence capture methods and next-generation sequencing (NGS) approaches have made feasible the analysis of the whole human exome. The exome deep sequencing, called whole-exome sequencing (WES) analysis, has already provided the opportunity to detect causal gene variants in dominant and recessive disorders [14], [15] and it has been recently proposed as a powerful tool to improve the molecular diagnostic of neonatal diabetes and maturity onset diabetes of the young (MODY) [16], [17].

Here, we applied a multi-step screening strategy aimed at identifying genes putatively related to CHI. This strategy included a TDT study on 17 patients and their families and exome sequencing in 10 CHI probands. We have also evaluated if NGS is a suitable alternative diagnostic approach to classical Sanger sequencing for CHI.

## Results and Discussion

To highlight novel CHI-associated genetic loci, we performed a multi-step screening strategy (Figure S1). First we carried out a genetic screening of *ABCC8*/*KCNJ11* genes on 33 CHI probands by classic sequencing approach; then, applying a genome wide SNP genotyping analysis on 17 non-consanguineous CHI patients (lacking *ABCC8*/*KCNJ11* mutations) and their families, we performed a TDT family-based association study, and finally we applied WES on 10 selected CHI probands (five from the TDT analysis group and five not subjected to previous molecular screening). To prioritize the coding variants identified by WES, we crossed the list of filtered WES variants with that comprising the TDT associated genes (51 genes), the known CHI-causative ones (10 genes), and a refined list of 211 CHI-functionally-related genes (see *Methods* for details).

### Whole-genome SNP Genotyping and TDT Analysis

The whole-genome SNP genotyping was performed on the 17 nuclear families. The overall results of the TDT association analysis are presented in the Manhattan plot (Figure S2). We obtained 144 SNPs associated at P≤0.005 (Table S1). None of these SNPs reached the stringent genome-wide significance of P<4.4×10^−7^; however, we considered any empirical P-value at 4.9×10^−5^ as “suggestive” (i.e. a potential reflection of an association signal) [18]. After genome annotation, we found that a subset of these SNPs map within/close to 51 genes (Table S1). Interestingly, we identified two SNPs located in the region of the *TLL1* (tolloid-like1) gene: rs3775321 (P = 0.000049; OR = 7.3, 95%CI = 2.2–24.5) and rs4691229 (P = 0.00043; OR = 0.2, 95%CI = 0.1–0.5). Using haplotype analysis we were able to confirm and to refine the potential association of the *TTL1* locus to the CHI (Table S2).

Applying the copy-number and homozygosity mapping analyses on 17 probands, we confirmed the absence of deletion or amplification events (*data not shown*); conversely, in two probands we found runs of homozygosity (ROH) longer than 2 Mb. The ROH segments were located on chromosome 4q24 (4.2 Mb containing 14 genes) in patient HI06, and on chromosome 6p36.33-q13.33 (7 Mb containing 26 genes) in patient HI18. By the subsequent WES analysis, we verified that all genes located in ROH segments were lacking mutations.

### WES Analysis and Gene Variants Identification

WES was performed on 10 CHI probands (Table 1). By sequencing the exome DNA libraries, we obtained 2.8–5.4 Gb of mapped sequence achieving a mean depth of target coverage between 27 and 50×, and an average of 19,181 variations per patient, including substitutions and indels (Table 2). Overall, the results of the percentage of target coverage at 10× were fairly homogeneous among samples (ranging from 83.5 to 88.7%). By looking to CHI known causative genes, we obtained at least 10× coverage in >95% of coding regions for *HADH*, *SLC16A1*, *UCP2*, *GLUD1*, and *KCNJ11*, whereas the values were slightly less uniform, reaching 90, 83, 80, and 75% for *INSR, ABCC8*, *GCK*, *HNF4A,* respectively. Only for *HNF1A* the percentage was low (55%). By focusing on non-synonymous single nucleotide variants (SNV, missense and nonsense) we counted an average of 9,395 variants per patient. After the filtering procedure, we counted an average of 430 variants (ranging from 368 to 494) that were not reported in publicly accessible databases (dbSNP132, 1000Genomes Project) and in our *in-house* database (built on 15 sequenced exomes, Italian individuals).

**Table 1:** Clinical characteristics of the ten CHI probands.

Proband	HI01P	HI06P	HI18P	HI26P	HI31P	HI37P	HI39P	HI42P	HI43P	HI44P
Sex	M	M	F	F	M	M	F	M	M	F
Macrosomia	No	No	No	No	Yes	No	No	Yes	Yes	No
Consanguinity	No	No	No	No	No	No	No	Yes	No	No
Onset	16 m	4 m	5 m	2 d	2 d	1 y	3 d	2 d	1 y	14 m
Clinical form	mild	severe	severe	severe	mild	mild	severe	severe	mild	severe
Pancreatectomy	No	No	Yes	No	No	No	No	Yes	No	No
Histology	No	No	Di-CHI	No	No	No	No	Di-CHI	No	No
TC PET	No	Fo-CHI	Di-CHI	No	No	No	Di-CHI	Di-CHI	No	Di-CHI
Response to Diazoxide	Yes*	Yes	No	Yes	Yes*	Yes	Yes§	No	Yes	Yes
Other metabolic features										
Leucine sensitive	No	No	Yes	No	No	No	No	No	No	No
Hyperammonemia	No	No	No	No	No	No	No	No	Yes	No
Mental retardation	No	No	No	No	No	No	No	No	Yes	No
Epilepsy	No	No	No	No	No	No	No	No	Yes	No
SNP array analysis	Yes	Yes	Yes	Yes	Yes	No	No	No	No	No

* Patient in Remission.

§ Medical Treatment with Diazoxide and Octreotide.

Abbreviations: m, month; d, day; y, year; Di, Diffuse; Fo Focal.

**Table 2:** Exome coverage and target coverage statistics of ten CHI probands.

CHI patients	HI01P	HI06P	HI18P	HI26P	HI31P	HI37P	HI39P	HI42P	HI43P	HI44P
***Whole exome sequencing results***										
Total number of raw reads (10^∧6^)	81.87	62.75	68.38	52.75	65.58	80.60	84.95	45.53	68.11	88.79
Total number of mapped reads (10^∧6^)	67.28	49.14	50.77	41.70	52.64	66.24	70.46	36.74	54.61	71.53
Target coverage (10×) (%)	88.67	87.38	88.00	86.46	88.40	88.51	88.36	83.55	88.43	88.16
Target coverage (20×) (%)	77.84	73.82	74.90	70.14	76.15	77.20	77.48	62.06	76.02	77.08
Mean read depth (x)	50.19	39.26	40.29	33.24	42.06	47.52	49.48	27.04	41.33	49.78
***Exonic SNV variants analysis***										
Total exonic SNP (N°)	19208	18751	19361	18959	19200	19697	19287	17808	20252	19293
Total exonic Indels (N°)	281	258	262	240	265	247	257	223	266	282
Missense and Nonsense SNPs (N°)	9436	9187	9461	9318	9456	9679	9433	8660	9890	9434
Filtering (dbSNP)	722	794	863	830	846	883	905	685	952	765
Filtering (1000g MAF<0.005)	618	705	761	721	737	778	800	608	825	652
Filtering (internal DB)	369	415	480	414	438	423	457	405	495	414

To prioritize the list of SNV obtained by WES, we used a list of 271 candidate genes based on three functional criteria of inclusion: the TDT associated genes (51 genes; Table S1), the known causative genes (10 genes), and 210 functionally-related genes (Table S3). Using this candidate gene list, we identified an average of 4.2 variants per patient which were then analyzed for their predicted impact on the encoded protein by predictor tools resulting in a final list of 27 single base variants distributed in 24 genes (Table 3). Among them, we found 24 missense mutations causing amino-acid changes predicted to be potentially damaging with high confidence by at least one prediction tool; we also identified two nonsense mutations and one splice site mutation. The mutations have been all confirmed by direct Sanger sequencing (*data not shown*).

**Table 3:** Whole-exome sequencing identification of non-synonymous SNP variants of 10 CHI patients.

Sample ID	Gene symbol	Chr	Position	RefSeq ID	Amino Acid change	PolyPhen/SIFT prediction
HI01	HNF1A	chr12	121426790	NM_000545	p.A161T*	*++/++*
	KCNH6	chr17	61615518	NM_030779	p.V532F	*−/++*
HI06	GNAS	chr20	57428464	NM_080425	p.E48D	*+/++*
	ACACB	chr12	109683531	NM_001093	p.N1760S	*−/++*
	NOTCH2	chr1	120464961	NM_024408	p.R1704H	*+/++*
HI18	SLC37A3	chr7	140035229	NM_032295	p.S389L	*−/++*
	CSMD1	chr8	3889535	NM_033225	p.R168W	*+/++*
	RYR3	chr15	33938601	NM_001036	p.T1272M	*+/++*
HI26	TRPV3	chr17	3417258	NM_145068	p.L776F	*++/++*
	TRPC5	chrX	111020098	NM_012471	p.G789R	*−/++*
	CAMK2D	chr4	114435066	NM_172115	p.R275C	*++/++*
HI31	PIK3R3	chr1	46521492	NM_003629	p.R306X	*Nonsense*
	CDKAL1	chr6	21231212	NM_017774	p.S561F	*−/++*
	SCN8A	chr12	52163721	NM_014191	p.V1148M	*−/++*
	KCNJ10	chr1	160011280	NM_002241	p.R348H	*−/++*
HI37	PDE4C	chr19	18331081	NM_000923	p.R253C	*++/++*
HI39	**ABCC8**	**chr11**	**17428964**	**NM_000352**	**p.Q953X** *	***Nonsense***
	**ABCC8**	**chr11**	**17418595**	**NM_000352**		***Splice site***
	NOS2	chr17	26107857	NM_000625	p.R314C	*++/++*
HI42	**ABCC8**	**chr11**	**17474673**	**NM_000352**	**p.A390E**	*−* ***/++***
	SLC24A6	chr12	113737647	NM_024959	p.Y564H	*++/++*
	SULF1	chr8	70501306	NM_015170	p.A222T	*−/++*
HI43	**GLUD1**	**chr10**	**88817449**	**NM_005271**	**p.S498L** *	*−* ***/*** *−*
	TLL1	chr4	166981332	NM_012464	p.G667S	*++/−*
HI44	CACNA1A	chr19	13325090	NM_001127222	p.R1966Q	*++/++*
	PC	chr11	66619274	NM_001040716	p.N657Y	*++/++*
	TLL1	chr4	166986893	NM_012464	p.H689P	*++/++*

* Mutation reported in Human Genome Mutation Database( HGMD).

PolyPhen/SIFT: (−) benign or tolerated; (+) possibly damaging/DAMAGING Low confidence;

(++) probably damaging/DAMAGING.

In bold known causative genes.

### Biological Relevance of Known and Novel CHI Gene Variants

By WES in 10 CHI patients we were able to identify four patients carrying mutations in three CHI causative genes (*ABCC8*, *GLUD1*, and *HNF1A*), seven patients showed genetic lesions in 17 genes implicated in the regulation of insulin secretion (*KCNH6, GNAS, ACACB, NOTCH2, RYR3, TRPV3, TRPC5, CAMK2D, PIK3R3, CDKAL1, SCN8A, KCNJ10, PDE4C, NOS2, SLC24A6, CACNA1A, PC*) and four patients carrying mutations in four genes suggested by the TDT analysis (*SLC37A3*, *CSMD1*, *SULF1*, *TLL1*). Overall, we identified: one patient displaying a unique variant in one gene, two patients are displaying mutations in two genes, and the remaining carrying multiple variants in more than two genes.

For known genes, we found a compound heterozygous patient carrying two mutations in *ABCC8*: a nonsense mutation pQ953X (CM981878) previously described [19] and a novel c.3989-2A>G splicing variant (patient HI39) abolishing the canonical acceptor splice site. We also identified a homozygous *ABCC8* variant (p.A390E) in exon 7 within a ROH on chromosome 11 (proband HI42) which has been previously reported in a case report [20]. The *GLUD1* mutation (pS498L,) was found at the heterozygous state in patient (HI43) who was affected by a mild CHI-HA (Hyperinsulinism-Hyperammoniemia) form and epilepsy. This mutation is associated to CHI-HA in the Human Gene Mutation Database (CM980942). Finally, we identified a *HNF1A* gene mutation (proband HI01) that has been previously reported in MODY3 patients [21]. Interestingly, the phenotype of our patient was similar to the one described by Stanescu, who recently reported mutations in the *HNF1A* gene in two cases of CHI [22].

For novel genes, by literature mining and database queries we have obtained indications about their biological relevance for pancreatic β-cell (Figure S3) and by gene ontology (GO) classification we group them in three main categories of cellular components such as transmembrane, cytosolic and mitochondrial proteins (Table 4).

**Table 4:** Functional annotation of novel variants.

GO cellular component	Relevant pathways ( KEGG)	Biological process in pancreas [ref]
*Transmembrane proteins*		
CACNA1A	Calcium signaling pathway, Type II diabetes mellitus	insulin exocytosis [23], [24]
CSMD1	undetermined	unknown
KCNH6	undetermined	regulating insulin secretion [25]
KCNJ10	Gastric acid secretion	unknown
NOTCH2	Notch signaling pathway	role in diabetes, MODY [16], [28]
RYR3	Calcium signaling pathway,	insulin secretion [32]
SCN8A	undetermined	ion permeability, insulin secretion [8], [34]
SLC37A3	undetermined	unknown
TRPC5,TRPV3	undetermined	ion permeability [9]
*Cytosolic proteins*		
ACACB	Fatty acid biosynthesis, Metabolic pathways, Insulin signaling pathway	insulin secretion [35], [36]
CAMK2D	Calcium signaling pathway,Wnt signaling pathway	insulin gene expression regulation [37]
CDKAL1	undetermined	insulin gene expression, insulin exocytosis [39]
GNAS	Calcium signaling pathway	β-cell proliferation, diabetes [41]
NOS2	Metabolic pathways, Calcium signaling pathway	β-cell proliferation, diabetes [43]
PDE4C	Purine metabolism	insulin secretion [45]
PIK3R3	Insulin signaling pathway, Type II diabetes mellitus	insulin signaling [47]
SULF1	undetermined	unknown
TLL1	undetermined	glucose regulation [49]
*Mitochondrial proteins*		
PC	Citrate cycle (TCA cycle), Pyruvate metabolism, Metabolic pathways	insulin secretion [10]
SLC24A6	undetermined	calcium homeostasis [51]

#### Transmembrane proteins

The *CACNA1A* gene encodes the alpha 1A subunit of the voltage-dependent (VDCC), P/Q type calcium channel. The protein is participating in the complex regulation of insulin secretion: the mechanism implicates that the closure of the K_ATP_ channels stimulates insulin secretion depolarizing the plasma membrane; the consequent opening of VDCC results in influx of extracellular Ca^2+^, which then triggers insulin exocytosis in pancreatic β-cells [23], [24]. *CSMD1* gene (belonging to the TDT analysis list) encodes an integral membrane protein with unknown molecular function. The *KCNH6* (alias *HERG2*) gene encodes a voltage-gated K^+^ channel (KV) belonging to the 6-TM family of potassium channels; interestingly; it has been described that HERG channels have a crucial role in regulating insulin secretion, raising the possibility that *HERG* mutations might be involved in some hyperinsulinemic diseases of unknown origin [25]. The *KCNJ10* gene encodes a member of the inward-rectifier potassium channel family (also known as 2-TM channels) and its mutations have been associated to SeSAME syndromes [26]. The *NOTCH2* gene encodes a type 1 transmembrane receptor that is expressed in the ductal cells during pancreatic organogenesis [27], and is belonging to the Notch family that has been suggested to have a role in diabetes [28]. Recently, a *NOTCH2* variant has been reported in one MODY affected patient [16], therefore similarly to other MODY causative genes [29]–[31], that are resulted also CHI causative, we propose *NOTCH2* as a good candidate for further investigations. The *RYR3* gene encodes for receptors that are intracellular ion channels expressed in human pancreatic β-cell. Interestingly, the activation of these receptors by ryanodine stimulates the insulin secretion at low glucose concentrations [32]. The *SLC37A3* gene (belonging to the TDT analysis list) encodes a transmembrane sugar transporters family, SLC37; to date, only the *SLC37A4* gene has been found mutated in the glycogen storage disease non-1A type [33]. The *SCN8A* gene encodes a member of the sodium channel alpha subunit gene family (Na_v_ 1.6), and it forms the ion pore region of the voltage-gated sodium channel and mediates the voltage-dependent sodium ion permeability of excitable membranes. Na_v_ 1.6 together with Na_v_ 1.7 (the other α subunit of human β-cells) is highly expressed in human islets [8], [34]. The *TRPV3* and *TRPC5 genes* encode proteins belonging to the transient receptor potential (TRP) cation channels group, they provide the background membrane conductance required for β-cells to depolarize upon K_ATP_ channel closure [9], but their role in human pancreas islets has not been studied.

#### Cytosolic proteins

The *ACACB* gene encodes a complex multifunctional enzyme system that catalyzes the carboxylation of acetyl-CoA to malonyl-CoA (the rate-limiting step in fatty acid synthesis); the malonyl-CoA can act as a coupling factor that regulates insulin secretion [35]. Interestingly, β-cell islets produce high quantity of ACACB enzyme [36]. The *CAMK2D*, encodes a protein Ca^2+^ sensing serine/threonine kinases (CaMK2) that it is involved in the regulation of Ca^2+^ mediated glucose-sensitivity of β-cells; in particular CaMK2 delta play a role as a feedback sensor, linking Ca^2+^ induced exocytosis to the re-synthesis of insulin acting on transcriptional regulation of genes related to the metabolic control of insulin secretion [37]. In humans, the CaMK2 beta and delta subtypes represent the major isoforms that are highly expressed in β-cells [38]. The *CDKAL1* gene, encodes a methylthiotransferase that modifies tRNA to enhance the translational fidelity of proinsulin transcript [39]. Moreover, a study using knockout mice for the *CDKAL1* gene revealed its possible role in controlling the first phase of insulin exocytosis in β-cells, through the K_ATP_ channel responsiveness [40]. The *GNAS* gene encodes the Gsα subunit of the G protein that is ubiquitously expressed. The encoded protein stimulates adenylyl cyclase (AC), thereby generating the second messenger cAMP. It has been shown that mice with β-cell-specific Gsα deficiency develop severe defect in β-cell proliferation and early onset insulin deficient diabetes [41]. The *GNAS locus* has a highly complex imprinted expression pattern and encodes four main transcripts: Gs-alpha, XLαs, NESP55, and the A/B transcript. Specifically, we found that our mutation is referred to the XLαs transcript, whose promoter is methylated on the maternal allele and transcriptionally active only on the paternal allele [42]. Therefore, for our proband, we hypothesize that a post-zygotic somatic event (such as methylation) and imprinting defect, resulting in the absence of expression of the maternal Gs-alpha isoform, might lead to the expression of mutated paternal allele in some affected focal area of the pancreas. The *NOS2* gene, has been implicated in β-cell damage and death in both type 1 and type 2 diabetes [43]; moreover, it mediates the IRS-2 expression contributing to β-cell failure [44]. The *PDE4C* gene encodes a protein belonging to the cyclic nucleotide phosphodiesterase subfamily PDE4. This protein hydrolyzes the second messenger, cAMP, thus playing a key role in many important physiological processes such as those mediated by hormones and neurotransmitters. It has been demonstrated that PDE1–4 enzymes are expressed in rodent pancreatic islets and β-cells [45] while PDE4C is the major isoform in human islets [46]. Furthermore, it has been shown that family-selective inhibition of PDE (PDE1, PDE3 and to some extent also PDE4) potentiates glucose-stimulated insulin secretion (GSIS); in particular, siRNA-mediated knockdown of PDE4C significantly enhanced GSIS in rat INS-1 (832/13) cells [45]. The *PIK3R3* encodes a family of lipid kinases that binds to activated (phosphorylated) protein-tyrosine kinases through the SH2 domain, regulates their kinase activity and triggers a plethora of intracellular events involving also the insulin signaling [47]. Notably, we found a mutation in the exon 7 that causes the complete loss of the second SH2 domain that may be deleterious to the protein function (Table 3). The *SULF1* gene (belonging to the TDT analysis list) encodes an enzyme with arylsulphatase activity acting on cell-surface heparan sulphate proteoglycans. It has been suggested an important role of this enzyme in pancreatic cancer progression [48]. The *TLL1* gene (belonging to the TDT analysis list) encodes an astacin-like zinc-dependent metalloprotease and is a subfamily member of the metzincin family; interestingly, it has been described as a gene responsive to a member of the NR4A subgroup of nuclear receptors that have been implicated in the regulation of glucose and lipid metabolism in insulin-sensitive tissues [49].

#### Mitochondrial proteins

The *PC* gene encodes the enzyme pyruvate carboxylase, which is a mitochondrial enzyme involved in gluconeogenesis, lipogenesis, insulin secretion, and synthesis of the neurotransmitter glutamate. PC seems to play an important role in the “amplifying signal” responsible for insulin secretion trough the K_ATP_ independent pathway [10]. Interestingly, Pineda et al. [50] reported an infant with what they termed the “French” type of pyruvate carboxylase deficiency, presenting initial neonatal symptoms such as respiratory distress, severe metabolic acidosis, and a tendency to hypoglycaemia. The *SLC24A6* gene encodes a mitochondrial Na^+^/Ca^2+^ exchanger (NCKX6) that maintain cellular calcium homeostasis and it plays a critical role in the mitochondrial Ca^2+^ transport in skeletal muscle, stomach, and pancreas [51].

### Conclusions

In the present investigation, we demonstrated that exome sequencing is useful as a diagnostic tool for CHI because identifies mutations in known genes but also gives the opportunity to discover potential damaging mutations affecting several genes combined in a complex fashion. WES, associated with family-based association analysis, revealed that CHI patients, lacking mutation in known causative genes, carry multiple exonic mutations. Due to the small number of patients of this study, some cautions should be taken into account to interpret the pathological role of identified variants. However, due to difficulties to collect large samples for such a rare disease, our results may be useful for future meta-analyses that will combine genetic data provided by other individual studies. The present study should be regarded as a starting point to design further investigations aimed from one side to the molecular characterization of the identified mutations, and from the other to investigate possible modifier genes, which might be relevant for the CHI pathogenesis. Increasing the number of well-characterized clinical CHI patients will allow the evaluation of the mutation frequency of novel gene variants and the identification of a clearer genotype-phenotype correlation.

Finally, we indicate that exome sequencing should be considered a valuable, time- and cost-effective diagnostic tool providing a fine molecular classification of CHI patient in alternative to Sanger sequencing, offering also the opportunity to better understand the genetic basis of CHI.

## Materials and Methods

### Study Sample and Strategy

The study was approved by the Ethical Committee of the San Raffaele Scientific Institute and performed according to the principles of the Declaration of Helsinki. Written informed consent was obtained from the next of kin on the behalf of the minors/children participants involved in this study. A detailed description of the study design is available through the Italian Registry of Congenital Hyperinsulinism (http://www.progettorici.it).

The diagnosis of CHI was carried out according to the following criteria: a fasting and post-prandial hypoglycemia with unsuppressed insulin secretion, a positive response to the administration of glucagon, negative ketone bodies in urine and plasma, and a prolonged dependence to treatment to prevent hypoglycemia. From the study we excluded infants from diabetic mothers, children with transient neonatal hyperinsulinism, with insulinoma, or Beckwith-Wiedemann Syndrome. Patients were considered to be diazoxide responsive if satisfactory glycemic control was achieved with doses of oral diazoxide not exceeding 20 mg/kg per day and were considered to be in remission if good glycemic control was maintain without further medical or surgical therapy. Patients were considered to be unresponsive to medical treatment if recurrent hypoglycemia episodes (<3 mmol/L) occurred during diet treatment (milk enriched with malto-dextrine or continuous enteral feeding) and treatment with diazoxide and octreotide at maximum dosage.

The study design is shown in Figure S1. We enrolled a total of 38 probands and their families; 33 probands were subjected to mutational screening of two “major” CHI genes (*ABCC8*, *KCNJ11*) by Sanger sequencing, following the procedure previously described [52]. After the mutational screening, 17 non consanguineous probands remained without a genetic diagnosis: together with their parents, they were hence processed for SNP genotyping and family-based TDT association analysis. Finally, we used WES analysis in 10 probands (for five of them, no mutational pre-screening was performed), either to find novel causative gene mutations or to evaluate WES as an alternative approach to classical Sanger sequencing. The clinical features of these patients are reported in Table 1.

### Whole-genome SNP Genotyping and Linkage Analysis

The whole-genome scan was performed by the Affymetrix GeneChip Human Mapping 250 K *Nsp*I Array Set (Affymetrix, Santa Clara, CA, USA), using standard protocols as described in the GeneChip® Mapping 500 K Assay Manual. Genotypes were called from cell intensity data by the BRLMM (Bayesian Robust Linear Model with Mahalanobis distance) algorithm implemented in the GeneChip Analysis Software v.4.0 (Affymetrix) using default parameter settings. SNP array raw data are available on Array Express repository (E-TABM-861). Copy number analysis and homozygosity mapping was carried out using CNAG 3.3, as previously described [53]. Allele and genotype frequencies were calculated for each *locus* and tested for Hardy-Weinberg equilibrium (HWE) considering only parents. For SNP quality control (QC), we employed predetermined QC inclusion criteria [minor allele frequency (MAF)>1%, SNP call rate >90%, HWE P>0.01]. The QC procedure also included a check for Mendelian errors (predetermined threshold: 1%), as well as sex and pedigree check. All procedures were performed by using the PLINK software [54], obtaining a set of 221,605 high-performing SNP markers for further analysis. A subset of SNPs that are in approximate linkage equilibrium with each other were hence selected by linkage disequilibrium (LD)-based pruning: 112,740 SNPs remained for family-based TDT association analysis.

We proceeded further performing the TDT association analysis on the 17 trios to search for association signals highlighting novel CHI candidate/modifier genes. Adjusted P-values were assessed by gene-dropping permutation, flipping the allele transmitted from parent to affected offspring, and using the default mode with the adaptative permutation approach. The maximum number of permutations per SNPs was 1,000,000. Considering the number of tests conducted, any empirical P-value at 4.4×10^−7^ would have been considered significant [55]; however, none of the SNPs reached the genome-wide significance. Hence, considering that: 1) the study cohort is obviously underpowered by its size; 2) “suggestive” P-values still may reflect evidence for association; and that 3) it is possible to make use of these suggestive P-values through the incorporation of prior biological knowledge; we decided to adopt P = 4.9×10^−5^ as significance threshold to build a SNP list to be used in all the subsequent analyses. All P-values are hence presented as not corrected.

Finally, we refined the association analysis on the *TLL1* gene performing a haplotype analysis. It was performed using all SNPs mapping in the *TLL1* genomic region and the sliding window specification option of the PLINK program (width of the window: 5 SNPs; shifting: 1 SNP at a time).

### WES Analysis

Three micrograms of genomic DNA was fragmented using the Covaris shearing system (Covaris inc., MA, USA). Exome capture was performed with the SureSelect Human All Exon Kit (v3, target size 50 Mb) according to the manufacturer’s instructions (Agilent technologies, Santa Clara, CA, USA). The platform design covers 1.22% of human genomic regions corresponding to the NCBI Consensus CDS Database (CCDS), including more than 700 human miRNAs from the Sanger v13 database and more than 300 additional human non-coding RNAs in a single tube. The selected coding regions were then massively sequenced by the Illumina GAIIx technology (Illumina, San Diego, CA, USA); following proprietary reversible terminator-based method and producing 2×76 bp read lengths. The paired-end sequence reads were mapped to the reference genome (UCSC NCBI37/hg19) using the Burrows-Wheeler Aligner (BWA) software [56] and the variant calling was performed using SAMtools [57]. The entire dataset of mapped reads (.bam files) is available on Sequence Read Archive, accession number ERP002635. The detected variations in each sample were filtered out using the minimum Phred SNP quality score of 100, the minimum depth of 10 reads, and the minor allele with an allelic fraction > 0.25. The variants were then annotated by the ANNOVAR software (http://www.openbioinformatics.org/annovar/annovar_db.html), for filtering common variants reported in dbSNP132 (http://www.ncbi.nlm.nih.gov), in 1000 Genomes Project (http://www.1000genomes.org/), and *in-house* database.

### Selection of Gene of Interest and Validation

For a focused evaluation of the whole-exome data, a list of 272 genes arising from three functional criteria of inclusion was used: ten genes known to be causative for CHI (*ABCC8*, *KCNJ11*, *GLUD1*, *GCK*, *HADH*, *HFN4A*, *SLC16A1*, *UCP2, INSR* and *HNF1A*); 51 genes suggested by TDT association analysis (Table S1); 145 genes associated to regulation of insulin secretion reported in the Rat Genome Database [58] and 66 genes indicated by reviewing the literature concerning the β-cell function and its metabolic regulation (Table S3).

To predict the effect of non-synonymous mutations on the encoded proteins of the selected gene list, we used two different web tools: SIFT [59] and PolyPhen v2.0 [60]. We reported non-synonymous mutations that were predicted deleterious at least by one predictor tool with a high score. To investigate the presence of functional interactions among genes, including direct (physical) and indirect (functional) associations, we used STRING, a database of known and predicted protein interactions [61]. Computer-assisted analysis for splice-site prediction was accomplished using the Neural Network Promoter Prediction Tool (NNPPT) program (http://www.fruitfly.org/seq_tools/splice.html) and the NetGene2 (release 2.4) program (http://www.cbs.dtu.dk/services/NetGene2).

All variants were validated by PCR amplification of exons carrying the identified mutation (*primers sequences and PCR conditions are available on request)* using genomic DNA from CHI probands. The amplified products were purified using montage the Micro PCR96 plates (Merck Millipore, Billerica, MA, USA) and then directly sequenced with the forward and reverse primers previously used for the amplification. Sequenced products were purified using the Montage SEQ 96 Sequencing Reaction Cleanup kit (Merck Millipore). Sequencing analysis was carried out using the Big Dye Terminator Cycle Sequencing Ready Reaction Kit v3.1 and an automated DNA sequencer (ABI-3100 XL Genetic Analyser; Applied Biosystems, Foster City, CA, USA). Factura and Sequence Navigator software Packages (Applied Biosystems) were used for mutation detection.

## Supporting Information

Figure S1 **Study design.** The initial study sample consisted of a total of 65 subjects, belonging to 32 CHI families. Overall 33 probands of these families were pre-screened for mutations in the two main CHI genes (*ABCC8,KCNJ11*). The 17 probands non consanguineous (lacking causal mutations in *ABCC8/KCNJ11)* together with their relatives were taken forward to whole-genome scan by the Affymetrix GeneChip Human Mapping 250 K Array. We applied TDT and haplotype analyses to highlight possible susceptibility/modifier CHI genes. Finally, 5 patients from the TDT study and 5 newly-enrolled CHI patients (not pre-screened for *ABCC8*/ *KCNJ11* were analyzed by whole-exome sequencing (WES).
(TIF)

Figure S2 **Manhattan plot.** Results of single-*locus* test of association between each of the 112,740 performing SNPs and CHI using the TDT association analysis. 112,740 single-marker permuted results (–log10 p-values) from the TDT test are plotted on each chromosome; red line represents P≤10^−3^ threshold, blue line represents P≤5×10^−3^ threshold.
(TIF)

Figure S3 **Schematic representation of insulin secretion in β-cell and the hypothetical role of novel CHI gene.** The entry of glucose in the β-cell trough the glucose transporter (GLUT2) stimulates insulin secretion by metabolic amplifying pathways producing ATP. The increase of ATP/ADP ratio leads to the closure of K_ATP_ channels, to the depolarization of the plasma membrane and to the subsequent activation of VDCC promoting influx of calcium into the cell. The overall modulation of the cytosolic free concentration [Ca^2+^] is essential for the triggering pathways of the insulin secretion. The binding of secreted insulin to its receptors (INSR), might activates the PI3K/Akt pathway and some transcription factors controlling insulin gene expression. Insulin exocytosis can also be influenced by neurotransmitters and hormones. Indeed, GLP1 actives AC leading the elevation of cAMP and the consequent PKA activation which finally mediates insulin exocytosis; alternatively the Ach mobilizes intracellular Ca^2+^ activating of IP3 receptor; then [Ca^2+^] binds to CaM activating CaMK and inducing the secretory process of insulin. Moreover, CDKAL1 is implicated in the control of the first phase of insulin exocytosis via K_ATP_ responsiveness. Other transmembrane ion channels might modulate electrical activity of the cellular membrane regulating the insulin secretion (KCN, TRP, SCN). Abbreviations: VDCC, voltage dependent calcium channel; TRP, transient receptor potential channels; KCN, potassium voltage-gated channel; SCN, sodium channel voltage-gated; ER, endoplasmic reticulum; SERCA, sarco/endoplasmic reticulum Ca^2+^ATPase; GIP, glucose-dependent insulinotropic peptide; AC, adenyl cyclase; GLP1, glucagon like peptide 1; INS, insulin; IRS1/2, Insulin receptor substrate 1/2; PLC, phospholipase C; IP3, Inositol trisphosphate; PKC, protein kinase C; DAG, diacylglycerol; Gs,Gi,Gq, G proteins; PKA, Protein kinase A; PI3K, phosphatidylinositol; CaM, calmodulin; Ach, acetylcholine; FA, fatty acid; FFA, free fatty acid. Yellow box indicate the CHI causative genes.
(TIF)

Table S1 **TDT data and associated alleles and genes.** 144 SNP resulted from TDT analysis at P≤0.005. OR, TDT odd ratio; CI L95 and CI U95, lower and upper 95% confidence interval for TDT odds ratio; Adjusted P-value, empirical p-value by adaptive procedure; A1/A2, A1: minor allele, A2: major allele; NP: number of permutations.
(XLS)

Table S2 **Haplotype analysis of TLL1 locus.**
(XLS)

Table S3 **Refined gene list.** The table is including 221 gene symbols and criteria of inclusion. PMID, pubmed identification number; RGD, rat genome database (http://rgd.mcw.edu/).
(XLS)
