# Supplementary figures and images for: Whole Genome SNP Genotyping and Exome Sequencing Reveal Novel Genetic Variants and Putative Causative Genes in Congenital Hyperinsulinism
Source: PLoS One. 2013 Jul 15;8(7):e68740. doi: 10.1371/journal.pone.0068740 (PMC3711910; doi:10.1371/journal.pone.0068740)

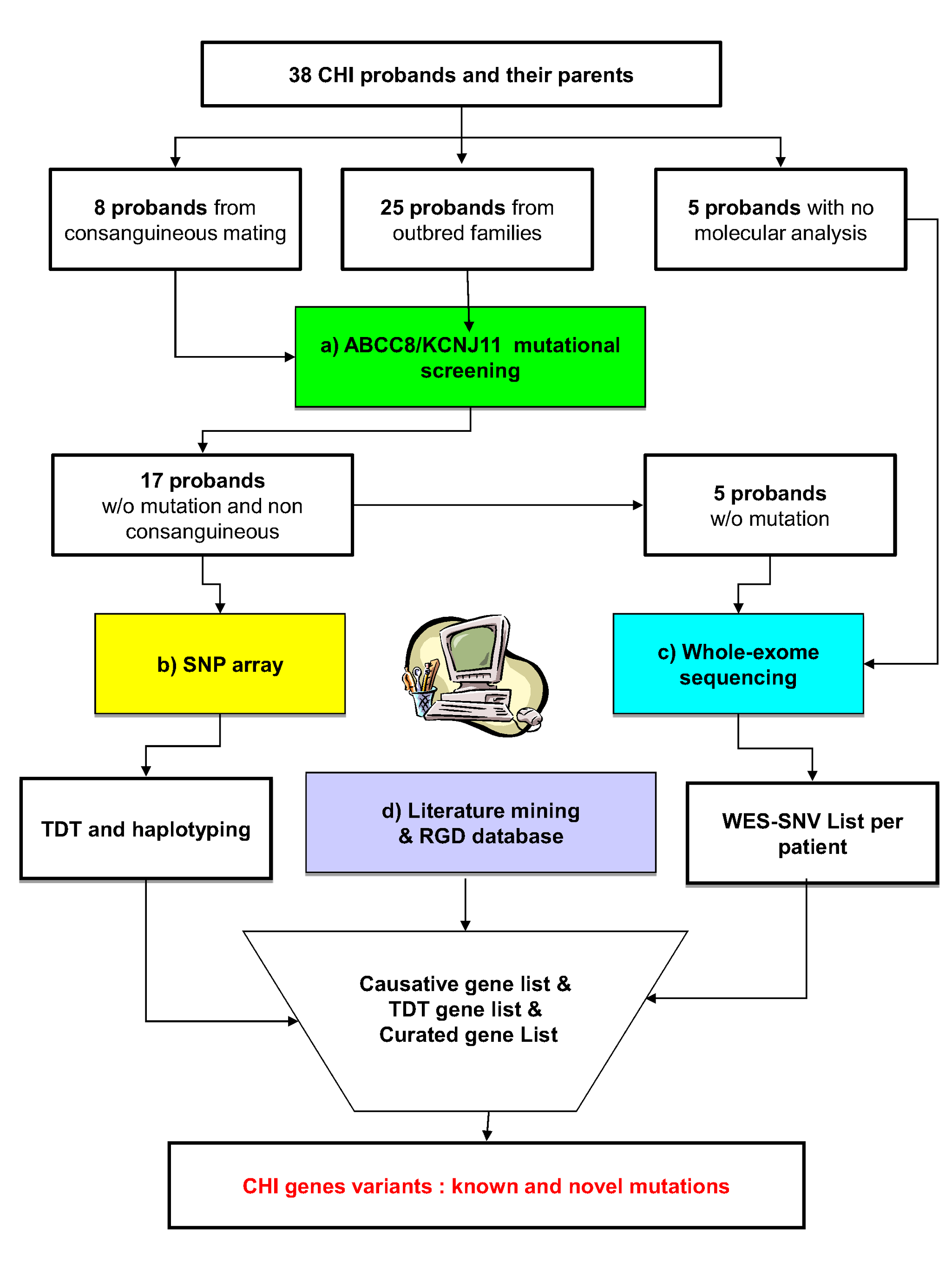

Supplement: Figure S1 — Study design. The initial study sample consisted of a total of 65 subjects, belonging to 32 CHI families. Overall 33 probands of these families were pre-screened for mutations in the two main CHI genes (ABCC8,KCNJ11). The 17 probands non consanguineous (lacking causal mutations in ABCC8/KCNJ11) together with their relatives were taken forward to whole-genome scan by the Affymetrix GeneChip Human Mapping 250 K Array. We applied TDT and haplotype analyses to highlight possible susceptibility/modifier CHI genes. Finally, 5 patients from the TDT study and 5 newly-enrolled CHI patients (not pre-screened for ABCC8/ KCNJ11 were analyzed by whole-exome sequencing (WES). (TIF) [file pone.0068740.s001.tif]

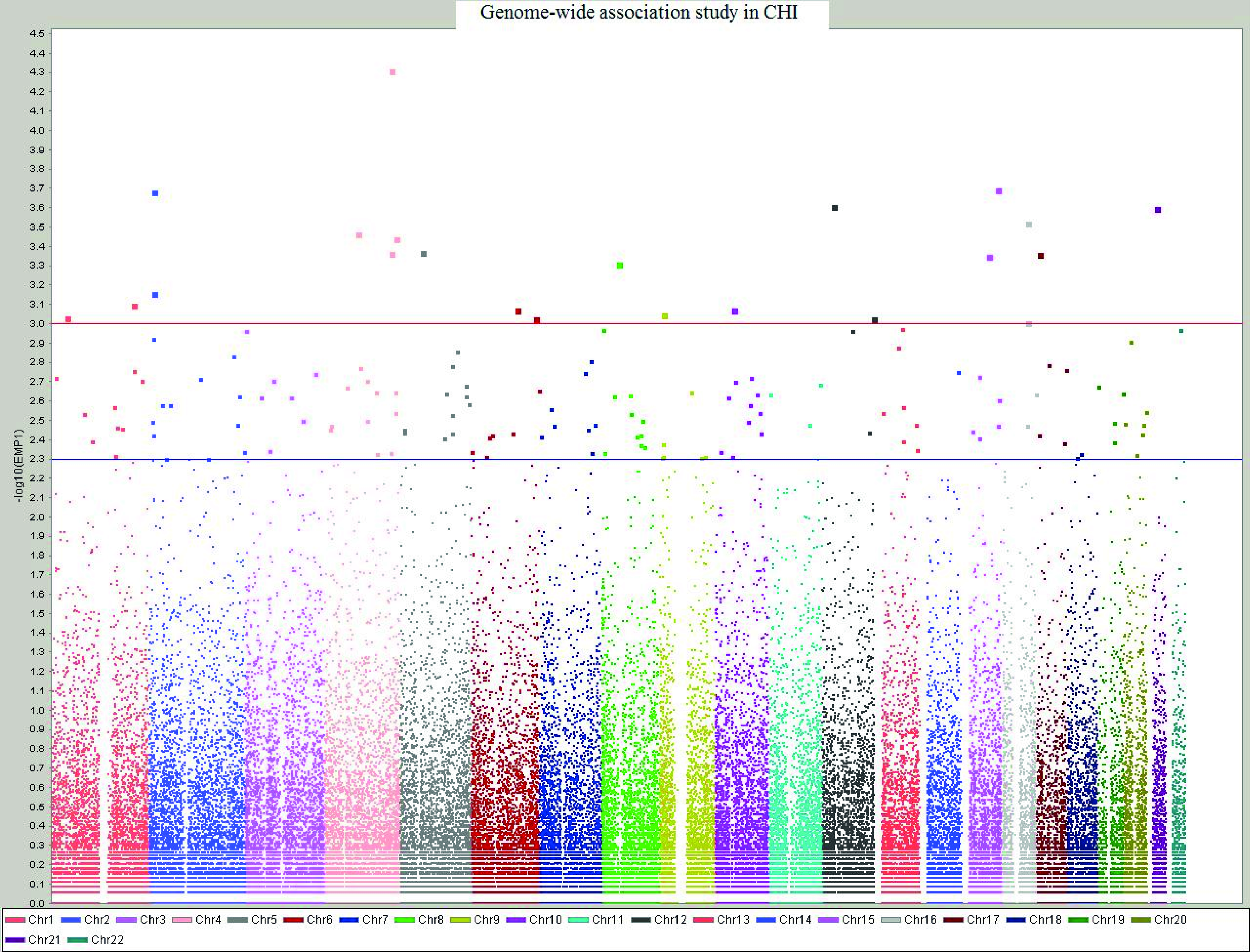

Supplement: Figure S2 — Manhattan plot. Results of single-locus test of association between each of the 112,740 performing SNPs and CHI using the TDT association analysis. 112,740 single-marker permuted results (–log10 p-values) from the TDT test are plotted on each chromosome; red line represents P≤10−3 threshold, blue line represents P≤5×10^−3^ threshold. (TIF) [file pone.0068740.s002.tif]

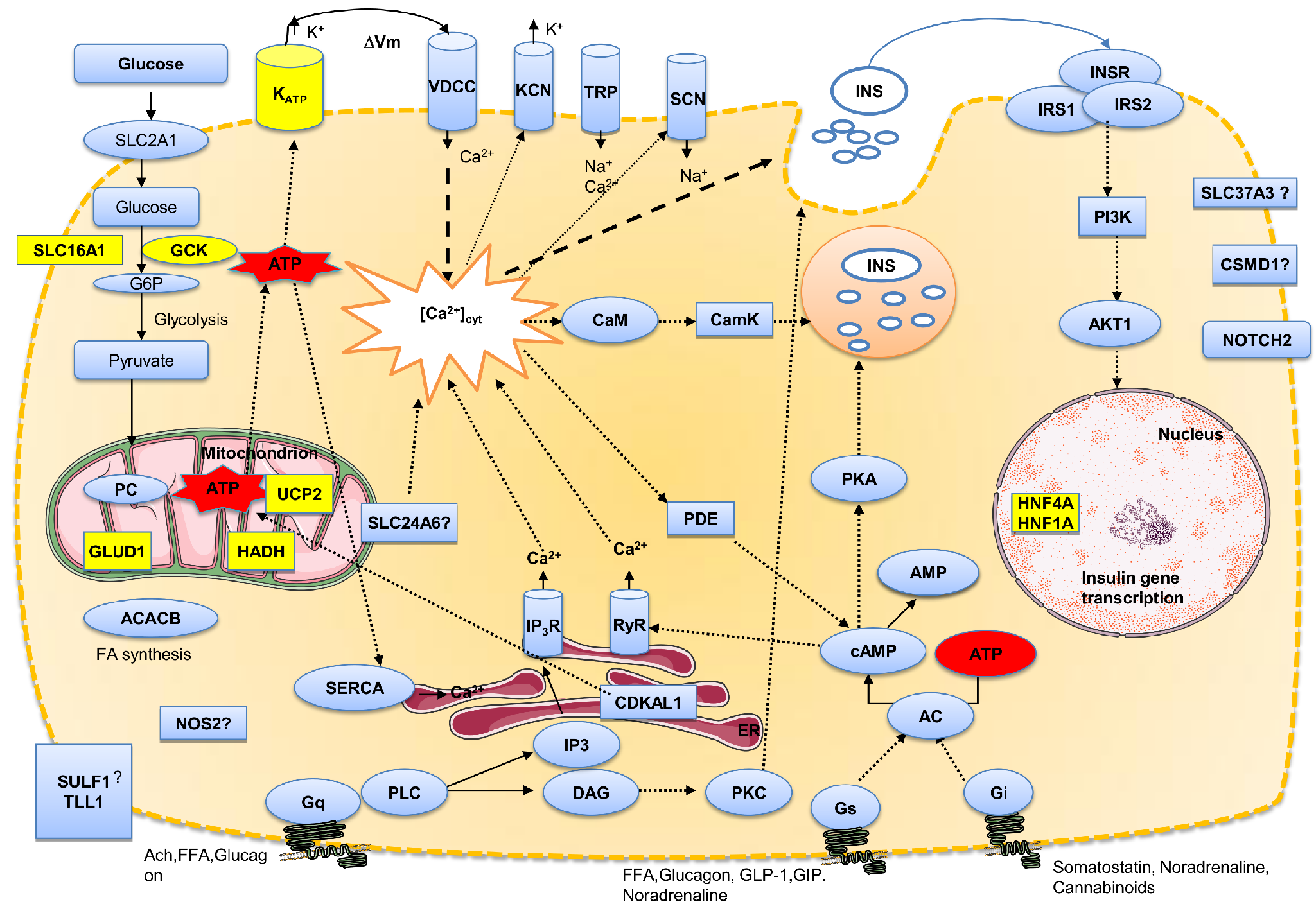

Supplement: Figure S3 — Schematic representation of insulin secretion in β-cell and the hypothetical role of novel CHI gene. The entry of glucose in the β-cell trough the glucose transporter (GLUT2) stimulates insulin secretion by metabolic amplifying pathways producing ATP. The increase of ATP/ADP ratio leads to the closure of KATP channels, to the depolarization of the plasma membrane and to the subsequent activation of VDCC promoting influx of calcium into the cell. The overall modulation of the cytosolic free concentration [Ca2+] is essential for the triggering pathways of the insulin secretion. The binding of secreted insulin to its receptors (INSR), might activates the PI3K/Akt pathway and some transcription factors controlling insulin gene expression. Insulin exocytosis can also be influenced by neurotransmitters and hormones. Indeed, GLP1 actives AC leading the elevation of cAMP and the consequent PKA activation which finally mediates insulin exocytosis; alternatively the Ach mobilizes intracellular Ca2+ activating of IP3 receptor; then [Ca2+] binds to CaM activating CaMK and inducing the secretory process of insulin. Moreover, CDKAL1 is implicated in the control of the first phase of insulin exocytosis via KATP responsiveness. Other transmembrane ion channels might modulate electrical activity of the cellular membrane regulating the insulin secretion (KCN, TRP, SCN). Abbreviations: VDCC, voltage dependent calcium channel; TRP, transient receptor potential channels; KCN, potassium voltage-gated channel; SCN, sodium channel voltage-gated; ER, endoplasmic reticulum; SERCA, sarco/endoplasmic reticulum Ca2+ATPase; GIP, glucose-dependent insulinotropic peptide; AC, adenyl cyclase; GLP1, glucagon like peptide 1; INS, insulin; IRS1/2, Insulin receptor substrate 1/2; PLC, phospholipase C; IP3, Inositol trisphosphate; PKC, protein kinase C; DAG, diacylglycerol; Gs,Gi,Gq, G proteins; PKA, Protein kinase A; PI3K, phosphatidylinositol; CaM, calmodulin; Ach, acetylcholine; FA, fatty acid; FFA, [file pone.0068740.s003.tif]
